# Analysis of the knowledge, attitudes, and practices of intensive care unit staff regarding *Candida auris*: a mixed-methods study in a private general hospital in Hanoi, Vietnam

**DOI:** 10.3389/fcimb.2026.1693788

**Published:** 2026-05-29

**Authors:** Lê Phan Khánh Huy, Saien Govender, Lê Minh Tiến, Mahomed Yunus Suleman Moosa, Andrew W. Taylor-Robinson

**Affiliations:** 1 College of Health Sciences, VinUniversity, Hanoi, Vietnam; 2 Centre for the AIDS Programme of Research in South Africa (CAPRISA), Durban, South Africa; 3 Nelson R. Mandela School of Medicine, University of KwaZulu-Natal, Durban, South Africa; 4 Department of Infectious Diseases, Nelson R. Mandela School of Medicine, University of KwaZulu-Natal, Durban, South Africa; 5 Center for Global Health, Perelman School of Medicine, University of Pennsylvania, Philadelphia, PA, United States

**Keywords:** antifungal stewardship, *Candida auris*, healthcare professional, infection control, intensive care unit, knowledge, attitudes and practices, survey, Vietnam

## Abstract

**Background:**

*Candida auris*, recently renamed *Candidozyma auris*, is an invasive fungal pathogen that is on the priority list on the World Health Organization (WHO) for future research. Infection poses major diagnostic and treatment challenges due to its phenotypic similarity to other *Candida* spp. and multiple antifungal resistance. Despite its emergence in Vietnam, the level of understanding among healthcare professionals is unknown. This study explores the knowledge, attitudes, and practices toward *C. auris* of registered physicians and nurses in a Vietnamese hospital intensive care unit (ICU).

**Methods:**

A mixed-methods study was conducted in an international standards-accredited Hanoi private general hospital. It comprised a self-administered cross-sectional survey containing 46 questions distributed to the ICU staff, followed by an in-depth semi-structured interview of selected participants. The survey covered demographic, knowledge, attitudes, and practices domains and utilized Likert-type and ranking scales. Descriptive and analytical statistics were applied to quantitative data and thematic analysis to qualitative insights.

**Results:**

A total of 32 ICU staff completed the survey, but only 11 (34.38%) scored above 50%. Knowledge was associated weakly with age and work experience but not gender or profession. Most participants recognized *C. auris* as an important ICU pathogen and expressed a positive attitude to institutional preparedness but revealed confusion about the site of infection, means of diagnosis, most effective agents for treatment, and best PPE practices to prevent infections. Of the 12 participants selected for in-depth interview, only two were familiar with the WHO guidelines on *C. auris*. The concerns centered on access to treatment, especially for immunocompromised patients. The opinions on outbreak likelihood varied, with some staff citing poor infection control in public hospitals as a risk, while others considered an outbreak unlikely due to low prevalence and the restricted mode of transmission. For multiple reasons, the private sector was perceived as better prepared than the public sector.

**Conclusions:**

This study reveals a knowledge gap regarding *C. auris* among ICU physicians and nurses in Hanoi caring for high-risk patient populations. The findings underscore a need for targeted continuing medical education, enhanced antifungal stewardship strategies, and revised infection control protocols to combat this clinically important emerging pathogen in Vietnamese hospitals.

## Introduction

*Candidozyma auris*, formerly known as *Candida auris*, is an emerging multidrug-resistant (MDR) fungal pathogen that is that in 2022 the World Health Organization (WHO) placed on its priority list for resarch, development, and public health action (WHO, 2022), with its importance reemphasized in 2024 (Casalini et al., 2024). Globally, more than 90% of clinical isolates are resistant to fluconazole (azoles), the most commonly available antifungal agent in Vietnam (Kwang et al., 2025), up to 15% are resistant to amphotericin B (polyenes), and approximately 1% are resistant to echinocandins (e.g., micafungin, caspofungin, and anidulafungin), the WHO-recommended first-line treatment for invasive infections (Ramage et al., 2026). Cases resistant to all of these three major antifungal classes have been reported, creating significant treatment difficulties and necessitating combination therapies. While single cases and outbreaks of *C. auris* have been noted across all WHO regions, its true global prevalence remains uncertain and is very likely underestimated due to diagnostic challenges (Rhodes and Fisher, 2019; Chen et al., 2020). Although reliable and rapid fungal diagnostics do exist, including mass spectrometry, polymerase chain reaction (PCR), and electrochemical detection, technical capacity and high cost typically render them inaccessible in low-resource healthcare settings (Kordalewska and Perlin, 2019; Mulet Bayona et al., 2020; Zhang et al., 2020; Alves et al., 2022).

With an overall mortality rate ranging from 30% to 72%, *C. auris* is an elusive pathogen that presents a growing public health risk (Thatchanamoorthy et al., 2022; Cabrera-Guerrero et al., 2025). The adhesin-specific ability of *C. auris* to associate with inert and biological surfaces, leading to persistent biofilm formation and colonization, poses a significant obstacle to infection control (Santana et al., 2023). Commonly used non-sporicidal disinfectants such as hydrogen peroxide, sodium hypochlorite, and quaternary ammonium compounds are relatively ineffective at removing all *Candida* spp. from medical devices (Cadnum et al., 2017), which underscores the imperative for healthcare professionals to understand the risks, practice effective prophylactic measures, and enforce strict infection control. This is particularly important in high-risk hospital wards with a large proportion of vulnerable patients, such as intensive care units (ICUs), where numerous *C. auris* outbreaks have been recorded (Eyre et al., 2018; de Almeida et al., 2021; Prestel et al., 2021). The healthcare system and patient demographic in Vietnam present several risk factors that make candidiasis a threat. These include high prevalence of hospital-acquired infections, widespread antimicrobial use, and an inpatient population with complex comorbidities (Phu et al., 2016).

Several nosocomial outbreaks of *C. auris* have been reported in South and East Asia (Chen et al., 2020; Kim et al., 2024; Cabrera-Guerrero et al., 2025), yet, to date, there has been no official report of a major outbreak in Vietnam nor a confirmed case in Hanoi, the location for this study. However, the fungus has been identified sporadically in the south of the country. First, in 2019, an 85-year old male visiting from Australia contracted a co-infection with another antifungal-resistant species, *C. duobushaemulonii*, which was diagnosed upon his medical repatriation to Melbourne (Xie et al., 2020). Subsequently, there have been five documented cases in female patients with COVID-19 admitted to a public hospital ICU in Ho Chi Minh City (Dang-Vu et al., 2024; Pham et al., 2024). Of these six reports of opportunistic *C. auris* candidemia, all in immunocompromised individuals with complex comorbidities, only one was not fatal.

As a lower-middle-income country, Vietnam faces several challenges to improve its fungal infection control capability. A survey of 24 public hospitals in 2023 revealed that although most (95.8%) fulfilled the basic requirements for laboratory testing facilities, only 66.7% performed antifungal susceptibility testing (AFST), while non-culture tests (such as β−D−glucan and PCR) were hardly utilized (Hieu et al., 2024). The absence of AFST in a hospital setting creates significant blind spots for infection prevention and control (IPC) and antimicrobial stewardship (AMS). Without AFST, clinicians are forced to rely on “blind” empiric therapy, which has direct consequences for both individual patient outcomes and the broader hospital microbiome.

In-country candidaemia was estimated in 2022 at a low rate of 12 cases per 100,000 population per annum, yet the incidence is rising compared to previous years. Considering the context of limited laboratory capacity and a lack of formal training in medical mycology for Vietnamese healthcare providers, this suggests that prevalence is underestimated (Duong et al., 2023). Recognizing the elusive nature of *C. auris* in traditional diagnostics places greater responsibility for the detection and management of this “superfungus” on non-designated clinical staff, including physicians and nurses, whose awareness and strict adherence to infection control principles play a critical role.

There is scant published literature on assessing the knowledge, attitudes, and practices (KAP) of healthcare providers globally regarding *C. auris*. One study in the Philippines found that while the hospital staff had satisfactory general knowledge and showed moderate concern for the emergence of *C. auris*, they lacked sufficient information to correctly identify the pathogen and treat infections (Sevilla et al., 2021). Another report from France showed that 30.6% of medical microbiologists nationwide knew the screening protocol for potential *C. auris* infection in their hospital, but only 8.3% of the facilities had appropriate tools to differentiate *C. auris* colonies (Guitard et al., 2024). Our broader literature search on candidiasis also did not capture the healthcare professionals’ specific awareness and practices toward this pathogen. The value of KAP studies such as this is that they provide a preliminary assessment of the preparedness of healthcare systems against emerging public health threats. In several studies, strict and immediate containment, surveillance, and proper training for staff were noted as key factors in the successful control of a *C. auris* outbreak (Sathyapalan et al., 2021; Nikopoulou et al., 2023; Rathod et al., 2025).

To our knowledge, despite the high rate of *C. auris*-associated mortality in ICUs, there is no literature on the understanding and perspectives of Vietnamese healthcare professionals treating vulnerable patients with severe underlying medical conditions. This mixed-methods study was designed to serve as an exploratory investigation to inform approaches to onsite and online continuing medical education (CME) in infectious disease control by determining the knowledge, attitudes, and practices regarding *C. auris* among ICU physicians and nurses. It aims to explore the perspectives of healthcare providers on an emerging global pathogen that is not yet prevalent where they practice but which carries major potential for fatal outbreaks.

## Materials and methods

### Study design

The study employed a mixed methodology, which consisted of two phases: (a) a survey and (b) an in-depth interview. The quantitative survey serves as a baseline assessment of the knowledge of healthcare professionals. The follow-up qualitative interviews were conducted to provide more details and context around the survey results, yielding a small yet meaningful insight into the on-ground experiences of ICU staff at a state-of-the-art hospital. Both instruments were conducted, transcribed, and analyzed in Vietnamese before being translated into English for reporting. Although both study phases were conducted in Vietnamese, the first language of all participants, most – including each interviewee – do have professional working proficiency and comprehension of medical terminology in English. The researchers involved in transcribing have bilingual proficiency in Vietnamese and English, a process overseen by native English speakers.

### Study population

All participants were current ICU staff at Vinmec Times City International Hospital, a private general hospital situated in Hai Bà Trưng District of Hanoi, the capital city of Vietnam. The hospital has a capacity of 600 beds, of which at any one time 3%–5% are in ICU, a low proportion that ensures a high nurse-to-patient ratio. It is the first medical center in Vietnam to be accredited by both the Joint Commission International Standards and the College of American Pathologists (Vinmec Healthcare System, 2026). This signifies a strong commitment to rigorous standards of safety and quality of care, establishing this urban hospital as a “best-case scenario” for assessing high-acuity patient KAP. This large teaching and training institute is recognized nationally as a pioneer in advanced medical procedures and patient therapies, which, in turn, demand a significant commitment and level of knowledge from its healthcare professional staff. A secondary consideration for study site selection was convenience based on proximity and access to the research team (the hospital is affiliated for undergraduate and graduate medical and nursing teaching purposes, too, and is within easy reach of VinUniversity).

All participants had to meet the following inclusion criteria: (1) aged 18 years or older, (2) willing and able to give informed consent, (3) spend at least 80% of their contracted working time in the ICU, (4) permanently employed or in residence training at the hospital for at least 2 months prior, and (5) currently registered as a practitioner with the Vietnam Ministry of Health. The study cohort was representative of all staff eligible to participate, and there was no indication of selection bias. The demographic characteristics of the 32 participants are shown in **Table 1**.

**Table 1:** Descriptive statistics of survey participants.

Sample characteristic (*N* = 32)	Response	
	*n*	%
Gender		
Male	18	56.25
Female	14	43.75
Age group (years)		
21–30	12	37.5
31–40	10	31.25
41–50	9	28.13
51+	1	3.13
Profession		
Physician	7	21.88
Resident doctor	10	31.25
Registered nurse	15	46.88
Work experience (years)		
≤ 2	9	28.13
2–5	4	12.5
6–10	4	12.5
> 10	15	46.88

### Cross-sectional survey

A descriptive cross-sectional survey, modified from a questionnaire validated in a similar study (Sevilla et al., 2021) and considered suitable to adapt for this setting, was administered to healthcare workers to evaluate their knowledge, attitudes, and practices toward *C. auris*. This was conducted via an anonymous, self-administered online platform (Google Forms) accessible via a QR code or a paper-based copy, based on participant preference. For the latter, responses were entered into the online platform and verified for accuracy so that all data were stored in a single password-protected repository. The survey period was from January 2024 to December 2025.

In preparing the study questionnaire, we sought to assess the knowledge ICU staff must have to adequately perform their duties. The two senior authors are a medical microbiologist and an infectious diseases physician, each with three decades of experience, with the latter working in ICUs in a range of settings. It is their expert opinion that most, but purposefully not all, questions fit within the scope of what one would expect of a well-trained frontline healthcare worker, be they a physician or a nurse. The survey consisted of a total of 46 items – nine questions to capture demographic information on each participant, plus 37 items which focused on three domains: knowledge (11 items), attitudes (10 items), and practices (16 items). Several questions on IPC were included to enable a baseline assessment of general knowledge of IPC, while others aimed to determine the depth of knowledge on *C. auris* as this influences adherence to protocols and communications.

For the first domain, the questionnaire used a combination of multiple-choice, single-best-answer, and ranking-choice questions to assess the participants’ awareness of general information, exposure risk, identification methods, and treatment options regarding *C. auris*. The knowledge score was calculated by assigning one point to each correct response, and the aggregate score was totaled (range, 0–11). The higher a participant’s score, the more their knowledge of *C. auris.* Based on a previously developed instrument (Sevilla et al., 2021), a nominal “pass” score of 6 was selected as the cutoff point (≥ 50% correct) for a satisfactory level of knowledge. For the two other domains, to capture the participants’ level of agreement to each item, the questionnaire employed a five-point Likert-type scale (1 = “completely disagree”, 2 = “disagree”, 3 = “neutral/no opinion”, 4 = “agree”, and 5 = “completely agree”) for attitudes and a four-point Likert-type scale (1 = “don’t know/unclear”, 2 = “unnecessary”, 3 = “necessary”, and 4 = “compulsory”) for practices. In healthcare, the term “necessary” implies competence and represents a functional requirement to achieve best patient outcomes, while the term “compulsory” implies compliance and is a mandatory requirement imposed by an external authority (such as a hospital’s regulations).

### Interview process

A total of 11 out of the 32 participants were conveniently selected and invited to participate in an in-depth interview. These comprised three consultant physicians and three senior nurses (each with > 10 years of ICU experience), plus three resident doctors and two junior nurses (each with ≤ 2 years of ICU experience). In addition, one medical microbiologist registrar from the study hospital’s Department of Microbiology was purposefully selected due to his expertise and referral by other participants. All 12 agreed and were interviewed individually in person by a trained researcher using a semi-structured format. The participants were given open-ended questions on their knowledge and concern of the emerging pathogen, current detection and treatment protocols, as well as their opinion on the institutional capacity and training programs to prevent or control a potential outbreak. Each interview lasted approximately 30 min and was voice-recorded with consent.

Each interview recording was transcribed verbatim and anonymized into an MS Word file by a researcher who was not involved with the original interviews. Field notes included non-verbal responses. Data were stored securely and analyzed thematically to identify patterns, themes, and contradictions in narratives. Two researchers overseen by an experienced supervisor worked independently on the dataset by coding themes and comparing notes before interpretation, considering existing literature and the quantitative results.

### Data analysis

Statistical analysis was performed using IBM SPSS Statistics (version 22, IBM Corp., 2018). All results of quantitative variables were reported either as mean (M), mode, median, standard deviation (SD), or frequency (*n*, %). Due to the relatively low number of samples (*N* = 32), the variables were analyzed using Fisher’s exact test on a 2 × 2 table to determine the relations between variables. In order to simplify demographic variables into two categories, age and work experience, the reported mean of the participants was used to determine a cutoff point: a confidence interval of 95% was used to determine statistical significance (*P* < 0.05).

## Results

### Demographic characteristics

A total of 32 participants were surveyed, 14 female (43.75%) and 18 male (56.25%), including ICU physicians (seven, 21.88%), resident doctors (10, 31.25%), and registered nurses (15, 46.88%) (**Table 1**). The age groups 21–30, 31–40, and 41–50 years old were approximately equally distributed, with the former (12, 37.5%) being slightly more represented. Nearly half of the participants (15, 46.88%) possessed over 10 years of professional experience.

### Phase 1: survey

**Knowledge:** Upon completion of the survey, only a minority (11, 34.38%) of the ICU staff participants achieved an average or higher score, which correlated with greater knowledge of *C. auris* (**Table 2**). The participants appeared to be most knowledgeable about the higher exposure risk of patients with previous antifungal treatment (30, 93.75%), followed by ICU being the common facility for *C. auris* patients (28, 87.5%) (**Appendix 1**). Misunderstandings occurred most often for questions on *C. auris* infection sites, for which only one participant answered all options correctly. Almost all (29, 90.63%) were not aware of the best laboratory test to detect and differentiate *C. auris*, with only three participants choosing the correct answer of “mass spectrometry”. Furthermore, most participants (24, 75.0%) held the belief that *C. auris* can be easily differentiated from other *Candida* species. In addition, the same high proportion (24, 75.0%) could not identify the most effective antifungal agents to treat *C. auris* infections. Other notable answer frequencies were recorded in **Appendices 2**-**5**. The knowledge score category varied by some demographic factors that were collected in the survey. While age and work experience were each weakly associated with knowledge (*P* = 0.042 and *P* = 0.048, respectively), neither gender nor healthcare profession were statistically significant (**Table 3**).

**Table 2:** Frequency of score category.

Score category (*N* = 32)	*N*	%
Below average (score < 6)	21	65.63
Above average (score ≥ 6)	11	34.38
M ± SD, 4.57 ± 1.92		

**Table 3:** Demographic determinants of knowledge score.

Determinant	Knowledge total score			
	Total	Score < 6	Score ≥ 6	*P*-value (two-sided), Fisher’s exact test
Age category				
< 40 years old	22	18	4	
≥ 40 years old	10	3	7	
				0.042
Gender				
Male	18	10	8	
Female	14	11	3	
				0.105
Profession				
Physician	17	9	8	
Nurse	15	12	3	
				0.072
Work experience				
< 10 years	17	14	3	
≥ 10 years	15	7	8	
				0.048

**Attitudes:** The participants perceived the threat of emergent *C. auris* infections as moderately high (*M* = 3.78, SD = 1.10) (**Appendix 6**). The responses also indicated positive attitudes toward institutional and personal preparation against potential *C. auris* infections and outbreak. Of these, the belief that *C. auris* requires special attention if detected scored the highest (*M* = 4.15, SD = 1.13), while most were unsure of the possibility that *C. auris* might be currently misdiagnosed/overlooked in their facility (*M* = 2.97, SD = 1.06) (**Appendix 6**). The same positive attitudes were apparent with the participants’ assessment of institutional capability to organize CME programs for healthcare providers on *C. auris*. While most were unsure if their workplace had previously run a CME program on *C. auris* (*M* = 3.25, SD = 0.72), the general consensus placed high confidence that their workplace can (*M* = 3.61, SD = 0.87) and will (*M* = 3.32, SD = 0.95) be able to organize appropriate training on the subject.

**Practices:** For general infection practices under the practices domain, all participants agreed on general infection control principles, specifically regarding handwashing (**Appendix 6**). This would include but not be limited to the use of common handwashing agents (alcohol-based rub, soap, and water), taking time for hand hygiene, and adequate access to hygiene supplies and equipment. Generally, the participants also believed that all listed personal protective equipment (PPE; gown, lab coat, disposable gloves, face shield, face mask, shoe covers, and hair net) are “necessary” but not “compulsory” during interaction with infected patients. The reported average for face shield and shoe covers were lower than for other PPE (both with *M* ± SD = 1.76 ± 0.75) (**Appendix 6**).

### Phase 2: follow-up interviews

Overall, the qualitative method revealed further that most participants did not have specific knowledge of *C. auris*, attributing this to both its lack of reporting in Vietnamese hospitals in which they had worked and a low global prevalence. Only two participants, the medical microbiologist and an early-career physician whose considerable knowledge identified them as an outlier, shared their awareness of the WHO research guidelines on the fungal pathogen and its call to prioritize monitoring. Another physician expressed serious concern about a *C. auris* outbreak happening in Vietnam, pointing out that “infection control [in Vietnam] is not yet optimal” and as such, “cross-infection and a subsequent epidemic is very likely to occur”. One pediatrics resident knew of *C. auris* reports in Ho Chi Minh City, saying “it’s only a matter of time before we see cases here. Perhaps [there] already have been but misidentified”. Nevertheless, many participants did not single out *C. auris* as a pathogen of concern, not distinguishing it from other *Candida* species and considering the treatment of candidiasis in general to be of similar priority to the management of other multi-resistant microorganisms of which they were familiar. One physician described the lack of effective drugs as a concern for *C. auris*: “Currently, some antifungal drugs are already available on the world market, but we do not have them in Vietnam”. Most participants agreed that treatment difficulty would be a major contributing factor to an outbreak in Vietnam, if it were to occur. A nurse cited concerns about infection control (hand hygiene, preventing cross-infection) due to insufficient resources and a high rate of fungal infections in Vietnamese public hospitals. In contrast, two physicians did not consider the pathogen a primary concern for now due to its current low prevalence in Vietnam and mode of transmission.

All ICU staff interviewed believed that an outbreak was unlikely to happen in the private hospital sector in Vietnam. Some attributed this perceived low risk to a good infection control and surveillance program or to the smaller number of patients being easier to handle, while others perceived that their hospital is actively monitoring for multiple fungi, including *C. auris*. Despite never encountering *C. auris* in their ICU, all participants were confident in their ability to manage the microorganism, with one physician saying, “The hospital management and department head all have a plan [in coordinating with infection control to manage a possible outbreak]”. The interviewees also mentioned the hospital’s infection control programs, guidelines, and PPE as factors contributing to their confidence. They shared that, in addition to adhering to standard quarantine principles, healthcare professionals in private hospitals regularly wash hands and wear PPE, supplies of which are frequently restocked. They added that such practices were not always available in public hospitals due to issues of overcrowding and underfunding. Annual training on infection control and CME programs on evolving pathogens are available, many participants shared, but as yet neither mention *C. auris*. Some nurses also perceived that education programs dedicated to specific pathogens would place an undue burden on their busy schedule, and instead they would prefer training based on mode of transmission. When asked about the capacity of their hospital to detect *C. auris*, while most participants admitted to not being fully aware of the current system, they expressed confidence in the ability of the department responsible. In particular, the microbiologist shared that “[Our hospital] currently has the technology to diagnose these [*Candida* fungi], specifically the MALDI-TOF MS…”. Overall, all healthcare professionals expressed confidence in their institution’s capacity and preparedness against potential outbreaks of any emerging pathogen, including *C. auris*.

There was no consensus among the participants on which area of improvement to focus, but they did share a common concern for the inequitable distribution of resources between sectors and hospital levels. For one physician, the problem of inequity may lead to decreased screening and diagnosis of *C. auris* and, in some cases, even to misdiagnosis. Consequently, patient volume has shifted toward advanced, often private, hospitals. Meanwhile, lower-tier public facilities remain underfunded and lack the specialized laboratory equipment necessary to distinguish between *Candida* species, significantly increasing the risk of misidentification. Other participants were interested in addressing the situation of overcrowded hospitals and their supply chain for PPE. Nurses, particularly, felt that overcrowding contributed greatly to public hospitals’ inability to completely comply with their own infection control regulations. Most healthcare professionals interviewed agreed that awareness of *C. auris* and other emerging pathogens should be improved. The point was articulated best by one of the consultant physicians:

“I think even though the infection rate is still low now, we still need to know about it. Second, I think it’s important to prepare in advance. If it happens, we are ready, we are proactive, we have the knowledge, we have the means, so we have enough human and material resources to control it, so that it doesn’t end up like the COVID pandemic. If you come unprepared, it will be very easy, it will very easily break out into a major pandemic and it will be very dangerous, so we need to prepare in advance—especially about spreading professional knowledge, learning about this disease and its causes of disease.”

## Discussion

The findings from the survey demonstrated insufficient knowledge among Vietnamese healthcare professionals regarding *C. auris*. This notion was corroborated by interviews, in which most participants admitted to not know much about *C. auris*. As such, it can be assumed that their low overall knowledge score stems from a lack of clinical cases and dedicated CME programs. The survey respondents attained high scores on risk assessment of patients for *C. auris* infection and on locating sites of infection but answered poorly on selecting the best laboratory test to identify the fungus. Specifically, 84.38% thought that this invasive fungus can cause skin infection (**Appendix 3**), which is unlikely according to existing literature (Alves et al., 2022; Thatchanamoorthy et al., 2022; Mallick et al., 2025). Most could answer correctly that common infection sites include the bloodstream and inside wounds. To identify *C. auris* by laboratory means, most responses (65.63%) selected traditional means of culture (**Appendix 2**), while recent literature considers mass spectrometry as the preferred option (Rhodes and Fisher, 2019; Alves et al., 2022). Similarly, 59.4% of the participants believed incorrectly, and 15.63% were unsure that *C. auris* can be easily differentiated from other *Candida* species by culturing (**Appendix 2**). These issues regarding identification are similar to those reported for Filipino healthcare providers (Sevilla et al., 2021). While ICU staff herein may, on occasion, suspect invasive candidiasis, all participants interviewed considered this outside of their remit and so referred responsibility for identification to the hospital’s Department of Microbiology, while physicians held responsibility for monitoring clinical symptoms.

Three-quarters of the survey respondents incorrectly identified the most effective antifungal agents for *C. auris* (**Appendix 1**), a critical finding with direct clinical implications for both prescribing practice and CME priorities in this setting, given the organism’s resistance profile and the challenges this poses for ICU management. When asked if their workplace CME program covers *C. auris*, most participants were uncertain, from which it can be assumed that the topic is either not covered or not covered sufficiently to provide any professional training value. However, the participants disagreed on the need to organize a specific CME program for *C. auris* because they believed that it would place too much attention on a pathogen that, while emerging in Vietnam, has yet to be reported in Hanoi. They instead preferred prioritizing the management of currently occurring pathogen groups, focusing primarily on Gram-negative bacteria, specifically carbapenem-resistant *Acinetobacter baumannii*, *Klebsiella pneumoniae*, and *Pseudomonas aeruginosa*, which are highly prevalent in Vietnamese ICUs (Toan et al., 2025). While such integrated IPC/AMS training is leading to improved patient outcomes in Vietnam (Doshi et al., 2025), the “diagnostic blindness” to *C. auris* reflects a more immediate focus on treatment and control rather than on surveillance and prevention of emerging threats. Although no association between knowledge and healthcare profession – physician or nurse – was found, this could not be refuted entirely due to the relatively low number of samples for generalizability. In this context, it is worth noting that a recent report revealed challenges in teaching healthcare-associated IPC to student nurses in Vietnam (Tuan Truong and PrevInf Group: Capacitating Asia’s Nursing Students on Innovative and Sustainable Prevention and Control of Healthcare-associated Infections, 2024).

Several of the survey questions on generic concepts of IPC could relate to any pathogenic microorganism spread by contact and so may be anticipated to be answered correctly by all categories of trained ICU staff. The specialized questions on microbiological differentiation and antifungal susceptibility aimed to assess the depth of awareness among frontline staff. Resident doctors in Vietnam undertake a 4-year general medical education program in pediatrics, internal medicine, or surgery, during which they spend 6–8 months full-time in ICU; all participating residents were surveyed at the completion of this period. While nurses are not expected to perform laboratory identification and AFST, their understanding of why a certain pathogen is unique can influence their adherence to specific IPC precautions and their communication with physicians, microbiologists, and medical laboratory technicians. The study results suggest that a *C. auris* risk profile has yet to be communicated effectively to ICU staff of all categories.

Interestingly, the majority of participants expressed high concern regarding the emergence of *C. auris* and for MDR microorganisms in general, similar to the findings in the Philippines (Sevilla et al., 2021). However, unlike their Filipino counterparts working in both public and private sectors, Vietnamese private healthcare professionals expressed a degree of confidence about their personal and institutional readiness to detect and manage the pathogen in their workplace. In the interview, most participants attributed their confidence to more readily available supplies, better equipment, fewer number of patients, effective AMS, and strictly enforced infection control protocols. The same certainty did not apply when asked if this extends to public hospitals since they cannot afford to maintain a similar level of resources devoted to stewardship and infection control.

A prominent issue that was pointed out is that Vietnamese hospitals are often exceeding capacity, a situation comparable to India, where patient overcrowding was indicated as underlying *C. auris* outbreaks (Rudramurthy et al., 2017). As such, there exists the likelihood that should a cluster of *C. auris* infections arise in Vietnam, the country’s public hospital settings are at similar risk for an outbreak to occur. Furthermore, in Asia’s tiered healthcare systems, the lower-level facilities that suffer from chronic underfunding of diagnostic infrastructure might not be sufficiently well-equipped to control the public health threat posed by this fungal pathogen (Denning, 2024). Even with the reassurances of the healthcare professionals interviewed here, an outbreak in the Vietnamese private hospital sector is a less likely yet still realistic scenario.

The decision to perform this study in a JCI-accredited hospital was deliberate, representing optimal conditions for IPC in Vietnam. The significant gaps in knowledge and understanding of *C. auris* identified in this environment are noteworthy in themselves and also because they imply that a hospital with fewer resources, such as in the public sector that serves the vast majority of the Vietnamese population, may be even less prepared for an outbreak. As such, these findings provide justification for a nationwide education program. In support of this, the interviewees’ reflections help shed light on the improvements required for Vietnam’s outbreak control strategy. The most popular suggestion is for more equitable distribution of resources between different levels and types of hospital, especially in terms of capacity and equipment (Quan and Taylor-Robinson, 2023). If lower-level public facilities can accurately identify cases (Denning, 2024), it would reduce the burden placed on higher-level ones, as well as on the smaller, private sector that is available to only the wealthiest members of Vietnamese society (Quan and Taylor-Robinson, 2023).

Another enhancement could be if the Ministry of Health were to release notifications to healthcare providers on *C. auris* and other emerging pathogens, especially those that similarly cause hard-to-treat infections in high-acuity patients. This would enable all hospitals in Vietnam to better track confirmed cases, to determine the risk of local emergence, and so to manage their outbreak preparedness plan. In this context, it should be noted that in November 2025, toward the end of conducting this study, the Vietnam Government approved a National Action Plan to Control and Prevent Antimicrobial Resistance in Human Health for the Period 2026–2030 (Ministry of Health, Vietnam Government, 2025). By embracing the One Health concept in recognizing the overuse and misuse of antibiotics in humans, livestock, and agriculture, this strategy document focuses squarely on combating the threat of AMR bacteria. However, targeting awareness by ICU staff of MDR fungal pathogens and a system that emphasizes requesting diagnostic tests are each notably absent.

### Limitations

Our study is limited by various factors that affect its generalizability. First, we recognize that the necessary modest sample size of 32 does not capture the scope of awareness of Vietnamese healthcare professionals. In mitigation, the cohort was similar to the 35 survey participants who took part in a comparable KAP study in the Philippines (Sevilla et al., 2021). Moreover, the one-center survey involved staff at an urban, private general hospital and thus lacked perspectives from other types of facility – notably in the public sector. Finally, the participants’ true knowledge of *C. auris* is difficult to determine as the survey included questions potentially answerable by reasonable assumption or educated guess based on commonly occurring related species such as *C. albicans* and *C. tropicalis*.

A professional biostatistician, whose main application is public health epidemiology, confirmed the suitability and rigor of the statistical analysis of the quantitative data. We consulted an experienced social scientist working in qualitative research methods, who was satisfied with how the interviews were conducted, data were collected and analyzed, and conclusions were drawn. The overall consistency of the responses indicated that for this subject-focused qualitative research the interviewee number was sufficient to identify major themes and approached data saturation for this homogeneous group of ICU staff. Both methodological experts emphasize that our findings are not invalidated by the perceived restriction of sample size and that the relevant data are sufficiently robust to support our discussion and conclusion.

We acknowledge that this study is exploratory in design, with the aim to pilot a methodology and generate hypotheses for further research. This should feature a diversity of hospital settings, assuming willingness to participate, and use questions to assess specific knowledge of *C. auris*.

## Conclusions

Amid the global emergence of MDR pathogens, *C. auris* is one of the hardest to treat and therefore potentially one of the most dangerous. Although only a few cases have been formally diagnosed in Vietnam so far. The possibility of a sudden outbreak remains high due to the nation’s proximity to outbreak-stricken countries with which it shares risk factors. This foundational study was conducted to determine the knowledge, attitudes, and practices toward *C. auris* of ICU physicians and nurses working in a Hanoi private hospital. Our findings suggest that knowledge of *C. auris* by Vietnamese healthcare professionals is limited, especially with regard to identification and differentiation. While the study participants were reasonably confident in the ability of their workplace to control and manage *C. auris*, insufficient knowledge and experience of clinical cases place this subjective assessment under scrutiny. Ergo, if this is not addressed by CME training of the ICU staff, future difficulty in treating *C. auris* may relate to the lack of knowledge on the best first-line treatment. Furthermore, perspectives on the infectious disease control capability of the lesser equipped public sector appeared quite negative, pointing to a pressing need for improvement, particularly in equitable resource management. We call for more extensive research into the looming threat of *C. auris* in Vietnam. This is in order to better prepare both the private and public healthcare systems against this MDR pathogen and to help design relevant trainings for Vietnamese healthcare professionals.

## Data Availability

The raw data supporting the conclusions of this article will be made available by the authors, without undue reservation.
