# Supplementary material for: Analysis of the knowledge, attitudes, and practices of intensive care unit staff regarding Candida auris: a mixed-methods study in a private general hospital in Hanoi, Vietnam
Source: Front Cell Infect Microbiol. 2026 May 29;16:1693788. doi: 10.3389/fcimb.2026.1693788 (PMC13261550; doi:10.3389/fcimb.2026.1693788)
Supplement: Supplementary file 1 [file Table1.pdf]

Appendix 1. Frequency of correct answers for the Knowledge domain

Question (N = 32)	Response: Answered correctly *n* (%)	Response: Answered incorrectly *n* (%)
**GENERAL INFORMATION**		
**Q1.** Which of these sites do *Candida auris* cause infection?	1 (3.13)	31 (96.88)
**Q2.** What is *Candida auris*?	21 (65.63)	11 (34.38)
**Q3.** Through which of the following can *Candida auris* be spread in the healthcare setting?	14 (43.75)	18 (56.25)
**EXPOSURE RISK**		
**Q4.** In which unit are patients most likely to have *Candida auris*?	27 (84.38)	5 (15.63)
**Q5.** Which of these are risk factors for *Candida auris* infection?	12 (37.5)	20 (62.5)
**Q6.** Fill in the blank: "Patients who have previously taken antifungal treatment are _________ to acquire *Candida auris* infection."	29 (90.63)	3 (9.38)
**Q7.** Ranking: How likely for each age group to be affected by *Candida auris*?	8 (25.0)	24 (75.0)
**IDENTIFICATION**		
**Q8.** What are the primary symptoms of *Candida auris* infection?	21 (65.63)	11 (34.38)
**Q9.** True / False: "*Candida auris* is easily distinguished from other *Candida* spp. on culture media".	8 (25.0)	24 (75.0)
**Q10.** What is the best laboratory test to identify *Candida auris*?	3 (9.38)	29 (90.63)
**TREATMENT**		
**Q11.** Ranking: What are the most effective antifungal agents against *Candida auris*?	8 (25.0)	24 (75.0)

Appendix 2. Participants' responses for single-answer questions under the Knowledge domain

Question (N = 32)		Response	
		n	%
	**EXPOSURE RISK**		
**Q4.**	In which unit are patients most likely to have *Candida auris*?		
	General medical ward	5	15.63
	General surgical ward	0	0
	Intensive Care Unit	27	84.38
	I don't know / I'm not sure	0	0
**Q6**.	Fill in the blank: "Patients who have previously taken antifungal treatment are _________ to acquire *Candida auris* infection."		
	More likely	29	90.63
	Equally likely	1	3.13
	Less likely	2	6.25
	I don't know / I'm not sure	0	0
	**IDENTIFICATION**		
**Q8.**	What are the primary symptoms of *Candida auris* infection?		
	Headache & neck stiffness	0	0
	Fever & chills	21	65.63
	Weight loss & fatigue	2	6.25
	Diarrhea & vomiting	9	28.13
	I don't know / I'm not sure	0	0
**Q9.**	True / False: "*Candida auris* is easily distinguished from other *Candida* spp. on culture media".		
	True	19	59.38
	False	8	25.0
	I don't know / I'm not sure	5	15.63
**Q10.**	What is the best laboratory test to identify *Candida auris*?		
	Mass spectrometry	3	9.38

Microscopical examination of cultures	21	65.63
Rapid antigen test	2	6.25
I don’t know / I’m not sure	6	18.75

Appendix 3. Participants' responses for multiple-answer questions under the Knowledge domain

Question (N = 32)	Response	
	Selected *n* (%)	Not selected *n* (%)
**GENERAL INFORMATION**		
**Q1.** Which of these sites do *Candida auris* cause infection?		
On the skin surface	27 (84.38)	5 (15.63)
In the bloodstream	31 (96.88)	1 (3.13)
In the muscle	3 (9.38)	29 (90.63)
In wounds	25 (78.13)	7 (21.88)
In the ears	13 (40.63)	19 (59.38)
I don't know / I'm not sure	0 (0)	32 (100.0)
**Q2.** What is *Candida auris*?		
A superficial skin colonizer	25 (78.13)	7 (21.88)
A common member of the gut microbiome	5 (15.63)	27 (84.38)
An invasive pathogen	28 (87.5)	4 (12.5)
I don't know / I'm not sure	0 (0)	32 (100.0)
**Q3.** Through which of the following can *Candida auris* be spread in the healthcare setting?		
Environmental surfaces	32 (100.0)	0 (0)
Inhaling aerosols (< 5 μm)	14 (43.75)	18 (56.25)
Drinking water	18 (56.25)	14 (43.75)
Hospital equipment	31 (96.88)	1 (3.13)
Ingestion of contaminated food	14 (43.75)	18 (56.25)
Contact by healthcare workers	32 (100.0)	0 (0)
Droplets spread (> 5 μm)	10 (31.25)	22 (68.75)
I don't know / I'm not sure	0 (0)	32 (100.0)
**EXPOSURE RISK**		

**Q5.** Which of these are risk factors for *Candida auris* infection?		
Total parenteral nutrition	22 (68.75)	10 (31.25)
Dialysis / Renal impairment	24 (75.0)	8 (25.0)
Central venous catheterization	24 (75.0)	8 (25.0)
Diabetes mellitus	26 (81.25)	6 (18.75)
Prolonged antibiotics treatment	32 (100.0)	0 (0)
< 10 days of hospital stay	5 (15.63)	27 (84.38)
HIV positive	30 (93.75)	2 (6.25)
Intubation / Mechanical ventilation	22 (68.75)	10 (31.25)
Chemotherapy	22 (68.75)	10 (31.25)
I don’t know / I’m not sure	0 (0)	32 (100.0)

Appendix 4. Participants' responses for Question 7 under the Knowledge domain

**Ranking: How likely for each age group to be affected by *Candida auris*?** (N = 32)	**Neonates** *n* (%)	**Children** *n* (%)	**Adults** *n* (%)	**Elderly** *n* (%)
Most likely	5 (15.63)	5 (15.63)	2 (6.25)	7 (21.88)
Likely	10 (31.25)	5 (15.63)	8 (25.0)	6 (18.75)
Less likely	3 (9.375)	12 (37.5)	10 (31.25)	7 (21.88)
Least likely	7 (21.88)	5 (15.63)	12 (37.5)	12 (37.5)
I don't know / I'm not sure	7 (21.88)	5 (15.63)	0 (0)	0 (0)

Appendix 5. Participants' responses for Question 11 under the Knowledge domain

**Ranking: What are the most effective antifungal agents against *Candida auris*?** (N = 32)	**Azoles** *n* (%)	**Echinocandin** *n* (%)	**Polyenes** *n* (%)
Most effective	10 (31.25)	15 (46.88)	1 (3.13)
Equally effective	5 (15.63)	5 (15.63)	14 (43.75)
Least effective	12 (37.5)	7 (21.88)	10 (31.25)
I don't know / I'm not sure	5 (15.63)	5 (15.63)	7 (21.88)

Appendix 6. Frequencies (median, mode, mean, SD) of Attitudes and Practices domains

	Variable	Range	Median	Mode	M ± SD
		Attitudes			
**Q1.**	I feel concerned about the emergence of *Candida auris* infections	1 – 5	4.00	4	3.78 ± 1.10
**Q2.**	I feel that my workplace would be able to manage an outbreak of *Candida auris* infections	1 – 5	4.00	4	3.64 ± 0.89
**Q3.**	I think *Candida auris* demands special attention if detected in my workplace	1 – 5	4.00	5	4.15 ± 1.13
**Q4.**	I'm confident I can manage *Candida auris* infection in a patient	1 – 5	4.00	4	3.48 ± 1.15
**Q5.**	I believe *Candida auris* infections at my workplace are being misdiagnosed / misidentified	1 – 5	3.00	3	2.97 ± 1.06
**Q6.**	I'm confident about the precautions to take when managing a patient with *Candida auris* infection	1 – 5	4.00	3	3.61 ± 0.87
**Q7.**	I'm confident that my workplace can organize CME program on *Candida auris* on demand	1 – 5	4.00	4	3.61 ± 0.87
**Q8.**	I believe my workplace had organized a CME program on *Candida auris* before	1 – 5	3.00	3	3.25 ± 0.72
**Q9.**	I'm confident that my workplace will have a CME program on *Candida auris*	1 – 5	4.00	4	3.32 ± 0.95

**Q10.**	I believe my workplace should have a CME program on *Candida auris*	1 – 5	4.00	4	3.49 ± 0.90
			**Practices**		
**Q1.**	I primarily use alcohol-based hand rub following contact with patients	1 – 5	4.00	4	4.40 ± 0.65
**Q2.**	I primarily wash my hands with soap and water following contact with patients with a confirmed infection	1 – 5	4.00	4	4.22 ± 0.74
**Q3.**	I primarily use both alcohol-based hand rub and wash my hands with soap and water following contact with patients	1 – 5	4.00	4	4.16 ± 0.70
**Q4.**	I wear disposable gloves for hand hygiene, especially in the absence of soap, water, and/or alcohol	1 – 5	4.00	4	3.66 ± 1.19
**Q5.**	I have access to well-maintained handwashing stations at or near the point of care	1 – 5	4.00	4	4.21 ± 0.62
**Q6.**	I have access to adequate hand hygiene supplies (e.g., gloves, hand soap or hand rub, disinfectants, etc.) at or near the point of care	1 – 5	4.00	4	4.21 ± 062
**Q7.**	I regularly perform hand hygiene before **AND** after contact with a patient	1 – 5	4.00	4	4.21 ± 0.62
**Q8.**	I perform hand hygiene before putting on and immediately after taking off gloves	1 – 5	4.00	4	4.12 ± 0.55
**Q9.**	Necessity of PPE: Gown	0 – 3	2.00	2	2.17 ± 0.58

**Q10.**	Necessity of PPE: Lab coat	0 – 3	2.00	2	2.17 ± 0.80
**Q11.**	Necessity of PPE: Disposable gloves	0 – 3	2.00	2	2.30 ± 0.49
**Q12.**	Necessity of PPE: Face shield	0 – 3	2.00	2	1.76 ± 0.75
**Q13.**	Necessity of PPE: Face mask	0 – 3	2.00	2	2.37 ± 0.53
**Q14.**	Necessity of PPE: Shoe covers	0 – 3	2.00	2	1.76 ± 0.75
**Q15.**	Necessity of PPE: Hair net	0 – 3	2.00	2	3.78 ± 1.10
**Q16.**	Frequency of disinfecting healthcare facility and equipment:<br>The healthcare facility at which I work is disinfected how often?	1 – 6	1.00	1	3.64 ± 0.89
